# Association of variably methylated tumour DNA regions with overall survival for invasive lobular breast cancer

**DOI:** 10.1186/s13148-020-00975-6

**Published:** 2021-01-18

**Authors:** Medha Suman, Pierre-Antoine Dugué, Ee Ming Wong, JiHoon Eric Joo, John L. Hopper, Tu Nguyen-Dumont, Graham G. Giles, Roger L. Milne, Catriona McLean, Melissa C. Southey

**Affiliations:** 1 Department of Clinical Pathology, Melbourne Medical School, The University of Melbourne, Melbourne, VIC 3010 Australia; 2 Precision Medicine, School of Clinical Sciences at Monash Health, Monash University, Clayton, VIC 3168 Australia; 3 Cancer Epidemiology Division, Cancer Council Victoria, Melbourne, VIC 3004 Australia; 4 Centre for Epidemiology and Biostatistics, School of Population and Global Health, The University of Melbourne, Melbourne, VIC 3010 Australia; 5 Anatomical Pathology, Alfred Health, The Alfred Hospital, Melbourne, VIC 3181 Australia

## Abstract

**Background:**

Tumour DNA methylation profiling has shown potential to refine disease subtyping and improve the diagnosis and prognosis prediction of breast cancer. However, limited data exist regarding invasive lobular breast cancer (ILBC). Here, we investigated the genome-wide variability of DNA methylation levels across ILBC tumours and assessed the association between methylation levels at the variably methylated regions and overall survival in women with ILBC.

**Methods:**

Tumour-enriched DNA was prepared by macrodissecting formalin-fixed paraffin embedded (FFPE) tumour tissue from 130 ILBCs diagnosed in the participants of the Melbourne Collaborative Cohort Study (MCCS). Genome-wide tumour DNA methylation was measured using the HumanMethylation 450K (HM450K) BeadChip array. Variably methylated regions (VMRs) were identified using the *DMRcate* package in R. Cox proportional hazards regression models were used to assess the association between methylation levels at the ten most significant VMRs and overall survival. Gene set enrichment analyses were undertaken using the web-based tool *Metaspace*. Replication of the VMR and survival analysis findings was examined using data retrieved from The Cancer Genome Atlas (TCGA) for 168 ILBC cases. We also examined the correlation between methylation and gene expression for the ten VMRs of interest using TCGA data.

**Results:**

We identified 2771 VMRs (*P* < 10^−8^) in ILBC tumours. The ten most variably methylated clusters were predominantly located in the promoter region of the genes: *ISM1*, *APC*, *TMEM101, ASCL2, NKX6, HIST3H2A/HIST3H2BB*, *HCG4P3, HES5, CELF2* and *EFCAB4B*. Higher methylation level at several of these VMRs showed an association with reduced overall survival in the MCCS. In TCGA, all associations were in the same direction, however stronger than in the MCCS. The pooled analysis of the MCCS and TCGA data showed that methylation at four of the ten genes was associated with reduced overall survival, independently of age and tumour stage; *APC:* Hazard Ratio (95% Confidence interval) per one-unit *M*-value increase: 1.18 (1.02–1.36), *TMEM101*: 1.23 (1.02–1.48), *HCG4P3*: 1.37 (1.05–1.79) and *CELF2*: 1.21 (1.02–1.43). A negative correlation was observed between methylation and gene expression for *CELF2* (*R* = − 0.25, *P* = 0.001), but not for *TMEM101* and *APC*.

**Conclusions:**

Our study identified regions showing greatest variability across the ILBC tumour genome and found methylation at several genes to potentially serve as a biomarker of survival for women with ILBC.

## Introduction

Invasive lobular breast cancer (ILBC) is the second most common histological subtype of breast cancer accounting for 10–15% of all cases [1–3]. ILBCs are typically oestrogen receptor (ER) and progesterone receptor (PR) positive and human epidermal growth factor receptor 2 (HER2) negative and are strongly associated with hormonal risk factors for breast cancer [4–7]. The incidence of ILBC increased sharply in the late 1990s as a consequence of the increased use of hormone replacement therapy (HRT) [8–13]. Awareness of the increased risk of breast cancer associated with HRT led to reduced use and a decline in ILBC incidence [14], but it has been shown to increase again recently [15, 16].

ILBCs display an obscure growth pattern with small, round and discohesive cells growing in a single file without forming any distinct clusters [17]. This is likely to be related to a loss of E-cadherin protein which is common in ILBC tumourigenesis and is a hallmark of this subtype [18]. Compared with other breast cancer types, ILBCs are less likely to form a firm and distinct lump and often present as undefined palpable masses on mammography [19, 20]. This poses a significant challenge for its early detection by routine mammographic screening [19, 21–23]. This occult nature may explain the detection of ILBC cases at advanced stages [4, 24–26]. ILBCs display a unique metastatic behaviour and often metastasis to the gastrointestinal tract [27, 28], colon [29], ovaries [30] and uterus [31], which is uncommon for other breast cancers types.

ILBC is biologically and histologically heterogeneous with several histological subtypes described that show distinct clinical behaviour and outcomes [3, 17, 32–35]. Aberrant tumour DNA methylation is a hallmark of cancer that occurs early in cancer development and is thus a potentially valuable marker of tumour progression and patient survival. Alterations in tumour DNA methylation have been investigated in detail for many types of cancer, including breast cancer but ILBCs are largely underrepresented in these studies [36, 37]. Studies focusing on ILBC-specific DNA methylation alterations have mainly used a candidate gene approach and have reported aberrant promoter methylation status for specific genes such as *CDH1* [38–41], *RASSF1A, HIN-1, RAR-β, cyclin-D2, TWIST* [42]*, ADAM33* [43], *SFRP1* [44] and *DAPK1* [45]. Moelans et al. (2015) compared the methylation profiles of classic ILBC (*n* = 20), pleomorphic ILBC (*n* = 16) and IDBC (*n* = 20) for 24 established and putative tumour suppressor genes and found lower *TP73* and *MLH1* promoter methylation and higher *RASSF1* promoter methylation in pleomorphic compared with classic ILBC [46]. Bae et al. (2004) compared the methylation profiles of ILBC (*n* = 19), IDBC (*n* = 60) and mucinous breast cancer (*n* = 30) for a panel of 12 genes and found *BRCA1* promoter hypermethylation in 92% of mucinous breast cancer compared with 39% in ILBC and 28% in IDBC. They also reported ILBC and mucinous breast cancer samples to be more frequently methylated for other genes in the panel compared with IDBC [47].

In this study, we hypothesised that genome-wide variations in DNA methylation patterns within the ILBC group may guide or reflect different tumour biologies leading to subgroups of tumours that differ in their clinical behaviour. Our aims were twofold: i) to investigate the genome-wide DNA methylation variability within the ILBC group and ii) to assess associations between tumour methylation at the most variable methylated regions and overall survival for women with ILBC.

## Results

### Study participants

The median age at breast cancer diagnosis in the MCCS was 65 years with tumours being diagnosed at stage 1A/1B (50%), 2A/2B (37%) and 3A/3C/4 (9%). There were 37 deaths observed during follow-up (median [IQR]: 13 [9–18] years). The tumours were mainly ER*-*positive, PR-positive and HER2-negative (47%). In TCGA data, the median age at diagnosis was 62 years. In both datasets, older women (aged 60 years or older at diagnosis) formed the majority of the cases (65%, in the MCCS and 58%, in TCGA). There was a higher proportion of young women at diagnosis (age less than 50 years: 21%) in TCGA compared with the MCCS (5%). The proportion of later-stage tumours (3A/3B/3C/4) was also higher in TCGA (33%) compared with the MCCS (9%). A total of 14 deaths were recorded during the follow-up (median [IQR]: 2 [1.5–5] years) in TCGA dataset. The clinical and pathological features of the study participants in the MCCS and TCGA and a comparison of the two studies are summarised in Table 1.

**Table 1:** Clinical and pathological features of the study participants from the MCCS and TCGA

Sample characteristics	MCCS *N* = 130	TCGA *N* = 168	*P* value
Median age at diagnosis (years), interquartile range	65 [25%; 58]	62 [25%; 51]	0.02
< 50 years (*n*, %)	6 (5)	35 (21)	0.0002
50–60 years (*n*, %)	39 (30)	35 (21)	
60+ years (*n*, %)	85 (65)	98 (58)	
Year of diagnosis (*n*, %)			
1992–1996	18 (14)	0 (0)	4.4 × 10^−30^
1997–2001	47 (36)	4 (2)	
2002–2005	36 (28)	15 (9)	
2006 and later	29 (22)	147 (86)	
Missing	0 (0)	2 (1)	
Overall deaths (*n*, %)	37 (28)	14 (8)	4.7 × 10^−06^
Median follow-up time (years)	13	2	2.2 × 10^−16^
Tumour grade (*n*, %)			
Grade I	13 (10)	NA	NA
Grade II	80 (61)	NA	
Grade III	17 (13)	NA	
Missing	20 (15)	NA	
Tumour stage (*n*, %)			
1A/1B	65 (50)	20 (12)	1.9 × 10^−12^
2A/2B	48 (37)	92 (55)	
3A/3C/4	17 (13)	55 (33)	
Missing	0 (0)	1 (0.5)	
Tumour ER expression (*n*, %)			
Positive	121 (93)	157 (93)	0.32
Negative	8 (6)	6 (4)	
Missing	1 (1)	5 (3)	
Tumour PR expression (*n*, %)			
Positive	94 (72)	140 (83)	0.004
Negative	35 (27)	22 (13)	
Missing	1 (1)	6 (4)	
Tumour HER2 expression (*n*, %)			
Positive	11 (8)	21 (13)	1.5 × 10^−5^
Negative	92 (71)	84 (50)	
Equivocal	5 (4)	35 (21)	
Missing	22 (17)	28 (17)	

*ER* oestrogen receptor, *PR* progesterone receptor, *HER2* human epidermal growth factor receptor 2

*P* = values are for chi-square tests and T-tests for categorical and continuous variables, respectively

### Variably methylated regions in ILBC

**Fig. 1:**
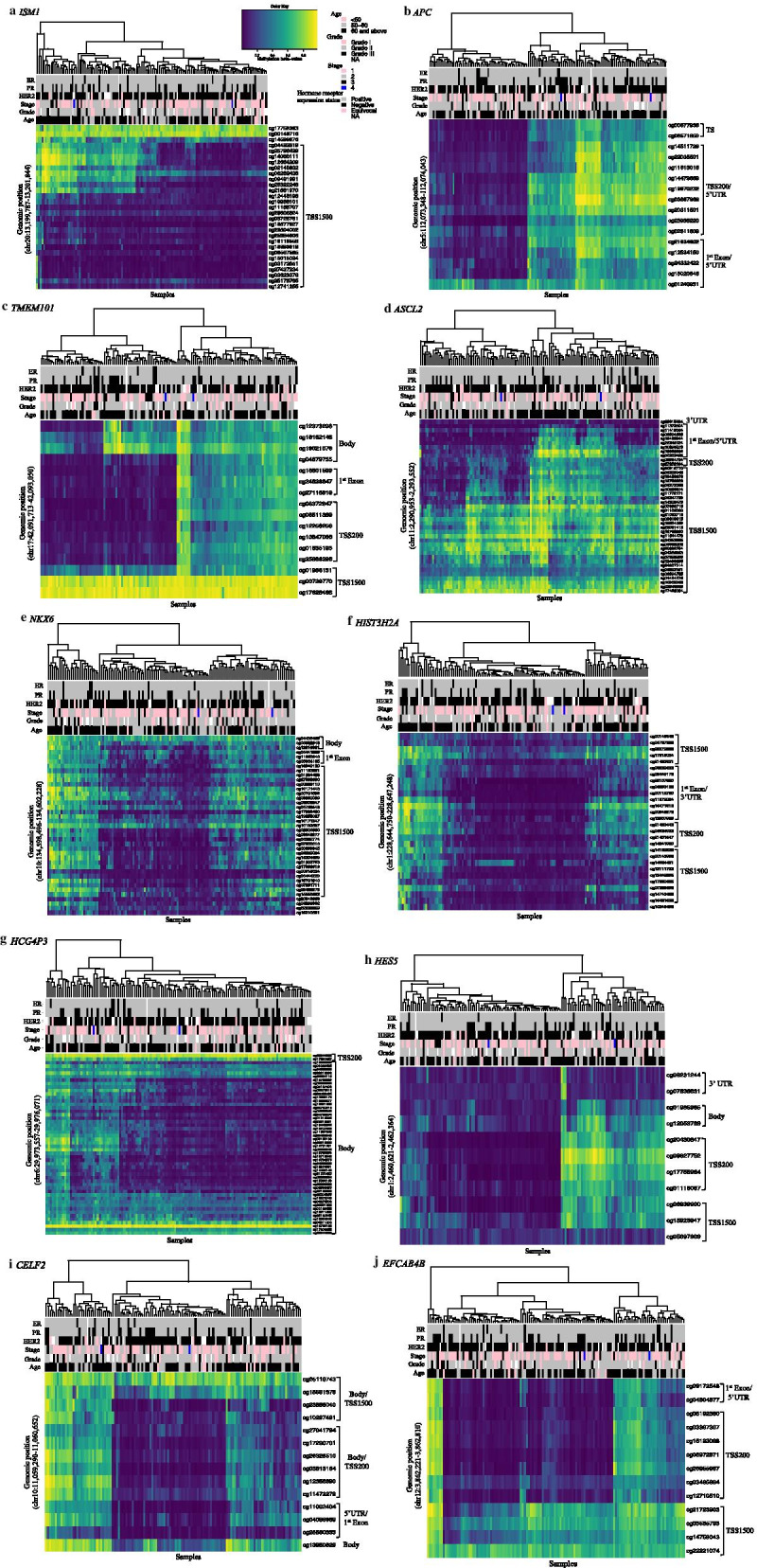
Methylation pattern of invasive lobular breast cancer (ILBC) samples. Heatmaps show the methylation patterns of invasive lobular breast cancer (ILBC) samples in the Melbourne Collaborative Cohort Study (MCCS) across the ten most significant variably methylated regions (VMRs): **a** *ISM1*, **b** *APC*, **c** *TMEM101*, **d** *ASCL2*, **e** *NKX6*, **f** *HIST3H2A*, **g** *HCG4P3*, **h** *HES5*, **i** *CELF2* **j** *EFCAB4B*. Annotation of CpGs by genomic position and location in the context of gene are marked on the maps. Annotation of samples by age at diagnosis and tumour characteristics are shown in the colour bars as indicated in the legend on the top-right. The methylation beta-value of the CpG positions shown in the heatmap is indicated in the colour key on the top-right corner

**Fig. 2:**
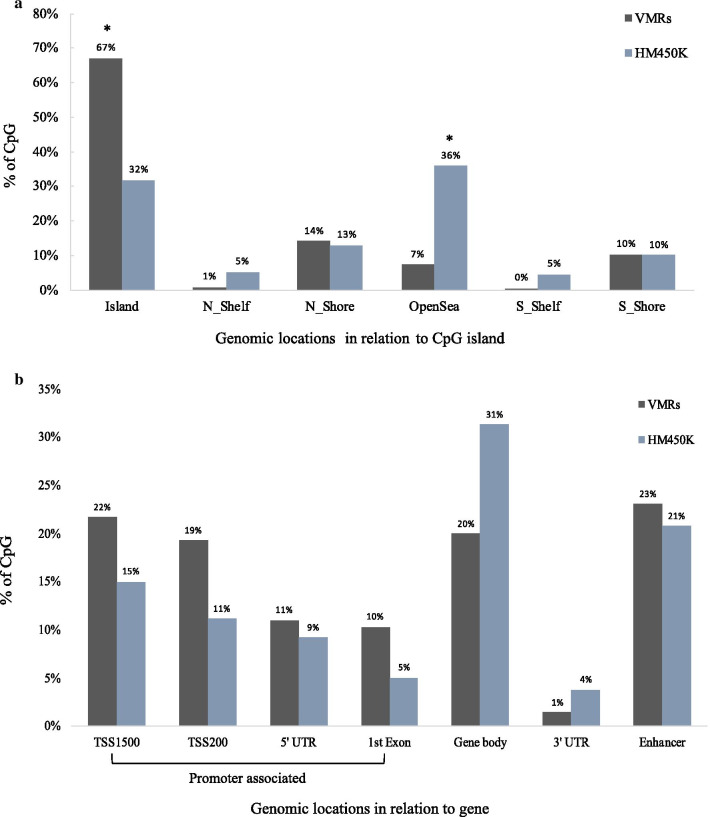
Genomic distribution of the variably methylated regions (VMRs). Bar plots show the distribution of 2771 variably methylated regions (VMRs) identified within invasive lobular breast cancer (ILBC) samples in the Melbourne Collaborative Cohort Study (MCCS) **a** relative to CpG islands, shores (0–2 kb from island), shelves (2–4 kb from island) and open sea and **b** in relation to the gene. Different genomic locations are shown on the x-axis and the percentage of CpG positions related to the VMRs is shown on the y-axis. The distribution of the HM450K probes relative to each CpG context is also indicated. *P*-values (Chi-square test) assessing significant enrichment in a given category relative to the HM450K array composition are indicated (*P < 0.001)

**Fig. 3:**
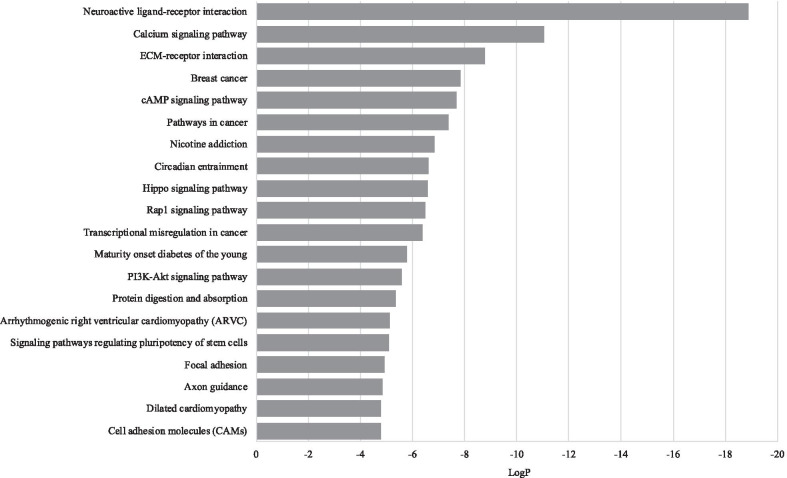
Twenty most significantly enriched KEGG pathways. Bar plot shows twenty most significantly enriched KEGG pathways in the variably methylated region (VMRs) identified within invasive lobular breast cancer (ILBC) samples in the Melbourne Collaborative Cohort Study (MCCS). The enriched terms are shown on the *y*-axis and the *P*-values (log transformed) assessing significant enrichment are shown on the *x*-axis

We identified 2,771 regions across the genome that showed substantially variable methylation (*P* < 10^−8^) across ILBCs in the MCCS (Additional file 2: Table S1). These VMRs corresponded to 2,208 genes and 563 intergenic regions. The most significant regions (*P* < 10^−8^) and the genes associated with these regions were chr20:13199787–13201844 (*ISM1,* 29 CpGs), chr5:112073348–112074043 (*APC*, 16 CpGs), chr17:42091713–42093050 (*TMEM101,* 16 CpGs), chr11:2290953–2293552 (*ASCL2,* 41 CpGs), chr10:134598496–134602228 (*NKX6*, 39 CpGs) and chr1:22844750–228647248 (*HIST3H2A/HIST3H2BB,* 28 CpGs). The average methylation level (beta-values) ranged between 0.09 and 0.63 at *ISM1,* 0.08 and 0.82 at *APC*, 0.15 and 0.83 at *TMEM101*, 0.15 and 0.77 at *ASCL2,* 0.07 and 0.70 at *NKX6,* and 0.05 and 0.58 at *HIST3H2A/ HIST3H2BB* (Fig. 1). There was some tendency for VMRs including more CpGs to be more highly ranked (Additional file 1: Fig. S1). We found a significant enrichment for CpG island-associated regions compared to all probes included in the HM450K array (Fig. 2a). Gene annotation also showed that 62% of the VMRs were located in gene promoter regions (1st Exon, 5 prime UTR, TSS1500 and TSS200) compared with 20% in gene body regions and 23% in enhancer regions (Fig. 2b). The pathway enrichment analysis showed that the genes associated with the VMRs were enriched for 1,973 terms (FDR-adjusted *P* < 0.05) including 54 KEGG pathways with stronger evidence for *neuroactive ligand-receptor interaction* (hsa04080), *breast cancer* (hsa05224), *pathways in cancer* (hsa05200), *hippo signalling pathway* (hsa04390), *Rap1 signalling pathway* (hsa04015) and *PI3K-Akt signalling pathway* (hsa04151). Figure 3 shows the twenty most significant KEGG pathways enriched in the VMRs.

Replication of the VMR analysis in TCGA dataset (*n* = 168), identified 2760 VMRs, of which 763 (28%) overlapped with the MCCS. The ten most significant VMRs identified in the MCCS ranked highly in the TCGA dataset (Table 2).

**Table 2:** Ten most significant VMRs identified in the MCCS and their respective ranking in TCGA

MCCS				TCGA		
Genomic location of the VMRs (GRCh37)	minfdr*	Number of CpGs	Associated gene	Genomic location of the VMRs in relation to the corresponding genes	minfdr*	Rank in TCGA
chr20:13199787–13201844	5 × 10^−181^	29	*ISM1*	TSS1500	1 × 10^−120^	10
chr5:112073348–112074043	5 × 10^−181^	16	*APC*	Body, 1st exon, TSS200, TSS1500	3 × 10^−170^	4
chr17:42091713–42093050	4 × 10^−172^	16	*TMEM101*	TSS1500, TSS200, 5′UTR, 1st exon	3 × 10^−92^	20
chr11:2290953–2293552	2 × 10^−152^	41	*ASCL2*	3′UTR, 1st exon, 5′UTR, TSS200, TSS1500	1 × 10^−90^	23
chr10:134598496–134602228	1 × 10^−142^	39	*NKX6*	Body, 1stExon, TSS1500	6 × 10^−118^	12
chr1:228644750–228647248	1 × 10^−131^	28	*HIST3H2A/HIST3H2BB*	TSS1500, TSS200	2 × 10^−196^	2
chr6:29973557–29976071	4 × 10^−124^	52	*HCG4P3/HLA-J*	Body	2 × 10^−194^	3
chr1:2460621–2462364	1 × 10^−110^	11	*HES5*	3′UTR, Body, TSS200, TSS1500	3 × 10^−90^	24
chr10:11059290–11060652	2 × 10^−109^	14	*CELF2*	TSS1500, TSS200, 5′UTR, 1st exon	9 × 10^−130^	7
chr12:3862221–3862810	6 × 10^−104^	13	*EFCAB4B*	1stExon, 5′UTR, TSS200, TSS1500	7 × 10^−198^	1

*minfdr: minimum adjusted *P*-value, TSS200 is the region from Transcript start site (TSS) to 200 nucleotides (nt) upstream of TSS; TSS1500 is the region from 200 to 1500 nt upstream of TSS; 5′ UTR is the region within 5 prime untranslated region, between the TSS and the ATG start site; body is the region between the ATG and stop codon; 3′ UTR is between the stop codon and poly A signal

Pathway enrichment analysis of the 763 overlapping VMRs resulted in 416 enriched functional terms (FDR-adjusted *P* < 0.05) including nine enriched KEGG pathways. Of these, 369 overlapped with pathways identified for all MCCS VMRs; *neuroactive ligand-receptor interaction* (hsa04080) and *hippo signalling pathway* (hsa04390) were among the KEGG pathways that were also found to be significantly enriched using all MCCS VMRs.

### VMRs and association with overall survival

In the MCCS, higher tumour methylation showed association with shorter overall survival for *APC* (HR = 1.28, 95% CI: 1.07–1.53), *HIST3H2A/HIST3H2BB* (HR = 1.28, 95% CI: 1.02–1.62), *CELF2* (HR = 1.30, 95% CI: 1.07–1.58) and *TMEM101* (HR = 1.21, 95% CI: 1.00–1.48). Weak evidence of association was also observed for *ISM1* (HR = 1.34, 95% CI: 0.97–1.85), *NKX6* (HR = 1.25, 95% CI: 0.98–1.60) and *HCG4P3* (HR = 1.24, 95% CI: 0.93–1.67). After adjusting for age at diagnosis and tumour stage, the association remained consistent for *APC* (HR = 1.24, 95% CI: 1.04–1.49), *TMEM101* (HR = 1.22, 95% CI: 0.99–1.51) and *HCG4P3* (HR = 1.25, 95% CI: 0.91–1.72) (Table 3). As shown in Table 3, all VMRs had an average methylation level below 0.5 and the direction of association was positive (gains in methylation associated with shorter survival).

**Table 3:** Hazard ratios (HRs) for the association between the methylation levels at the ten most significant variably methylated regions (VMRs) and overall survival in the Melbourne Collaborative Cohort Study (MCCS) and The Cancer Genome Atlas (TCGA) dataset

MCCS								TCGA						
				Adjusted for age		Adjusted for age and stage					Adjusted for age		Adjusted for age and stage	
Gene*	Averagemethylation**	HR (95% CI)	*P*	HR (95% CI)	*P*	HR (95% CI)	*P*	Average methylation**	HR (95% CI)	*P*	HR (95% CI)	*P*	HR (95% CI)	*P*
*APC*	39.1	1.28 (1.07–1.53)	0.01	1.24 (1.04–1.47)	0.02	1.24 (1.04–1.49)	0.01	35.9	1.16 (0.89–1.51)	0.28	1.12 (0.86–1.44)	0.41	1.06 (0.82–1.38)	0.63
*TMEM101*	39.6	1.21 (1.00–1.48)	0.06	1.19 (0.98–1.44)	0.08	1.22 (0.99–1.51)	0.06	39	1.13 (0.77–1.66)	0.52	1.12 (0.78–1.60)	0.54	1.27 (0.87–1.85)	0.21
*ISM1*	22.8	1.34 (0.97–1.85)	0.07	1.02 (0.76–1.38)	0.89	0.90 (0.65–1.26)	0.54	19.2	1.48 (0.91–2.41)	0.11	1.38 (0.80–2.37)	0.25	1.48 (0.86–2.54)	0.15
*ASCL2*	44.4	1.16 (0.81–1.65)	0.42	0.93 (0.66–1.31)	0.69	0.99 (0.71–1.38)	0.95	44.6	1.28 (0.74–2.20)	0.38	1.17 (0.68–2.02)	0.57	1.44 (0.81–2.57)	0.22
*HIST3H2A*	20.1	1.28 (1.02–1.62)	0.03	1.08 (0.86–1.35)	0.53	1.03 (0.82–1.29)	0.78	20.1	1.35 (1.00–1.83)	0.05	1.28 (0.94–1.73)	0.12	1.23 (0.90–1.68)	0.18
*NKX6*	29	1.25 (0.98–1.60)	0.07	1.06 (0.83–1.35)	0.63	1.01 (0.79–1.29)	0.91	30.2	2.06 (1.32–3.21)	0.001	1.88 (1.21–2.92)	0.01	2.01 (1.28–3.17)	0.002
*HCG4P3*	29.1	1.24 (0.93–1.67)	0.14	1.13 (0.84–1.53)	0.41	1.25 (0.91–1.72)	0.16	31.1	2.04 (1.32–3.15)	0.001	1.80 (1.13–2.85)	0.01	1.69 (1.05–2.72)	0.03
*HES5*	19	1.11 (0.88–1.40)	0.38	1.11 (0.88–1.40)	0.37	1.13 (0.89–1.42)	0.29	20.9	1.26 (0.88–1.80)	0.21	1.15 (0.80–1.65)	0.45	1.13 (0.76–1.68)	0.53
*CELF2*	33.4	1.30 (1.07–1.58)	0.01	1.12 (0.93–1.36)	0.23	1.13 (0.93–1.36)	0.21	35	1.50 (1.06–2.12)	0.02	1.44 (1.01–2.05)	0.04	1.51 (1.07–2.13)	0.02
*EFCAB4B*	32.8	1.01 (0.83–1.23)	0.88	0.96 (0.80–1.15)	0.63	0.99 (0.83–1.19)	0.99	34.5	1.41 (1.05–1.89)	0.02	1.32 (0.98–1.78)	0.07	1.25 (0.93–1.67)	0.14

*Gene: Gene associated with the variably methylated regions (VMRs), most of the VMRs were located in the promoter region of the genes, **Average methylation: Average methylation level (beta-value) of the samples across the VMRs, *HR* hazard ratio, *CI* confidence interval

In TCGA dataset, the crude HRs were all positive, consistent with the MCCS dataset, albeit generally greater, in particular for *ISM1* (HR = 1.48, 95% CI: 0.91–2.41), *ASCL2* (HR = 1.28, 95% CI: 0.74–2.20), *NKX6* (HR = 2.06, 95% CI: 1.32–3.21), *HIST3H2A/HIST3H2BB* (HR = 1.35, 95% CI: 1.00–1.83), *HCG4P3* (HR = 2.04, 95% CI: 1.32–3.15), *CELF2* (HR = 1.50, 95% CI: 1.06–2.12) and *EFCAB4B* (HR = 1.41, 95% CI: 1.05–1.89). Associations remained consistent after adjustment for age at diagnosis and tumour stage for all VMRs except those located at *APC* and *HES5*. The pooled HRs after adjustment for age at diagnosis and tumour stage showed that methylation was associated with overall survival for four genes: *APC* (HR = 1.18, 95% CI: 1.02–1.36), *TMEM101* (HR = 1.23, 95% CI: 1.02–1.48), *HCG4P3* (HR = 1.37, 95% CI: 1.05–1.79) and *CELF2* (HR = 1.21, 95% CI: 1.02–1.43) (Table 4).

**Table 4:** Pooled hazard ratios for the association between methylation levels at the ten most VMRs and overall survival: meta-analysis of the MCCS and TCGA results

			Adjusted for age		Adjusted for age and stage	
Gene*	HR (95% CI)	*P*	HR (95% CI)	*P*	HR (95% CI)	*P*
*APC*	1.24 (1.07–1.44)	0.004	1.20 (1.04–1.39)	0.01	1.18 (1.02–1.36)	0.03
*TMEM101*	1.19 (1.00–1.42)	0.05	1.17 (0.99–1.39)	0.06	1.23 (1.02–1.48)	0.03
*ISM1*	1.38 (1.05–1.80)	0.02	1.09 (0.84–1.42)	0.50	1.03 (0.77–1.36)	0.83
*ASCL2*	1.19 (0.88–1.61)	0.24	0.99 (0.74–1.33)	0.96	1.08 (0.81–1.45)	0.57
*HIST3H2A*	1.30 (1.08–1.57)	0.004	1.15 (0.95–1.37)	0.14	1.09 (0.91–1.31)	0.33
*NKX6*	1.40 (1.13–1.74)	0.002	1.21 (0.98–1.50)	0.08	1.18 (0.95–1.46)	0.13
*HCG4P3*	1.45 (1.13–1.85)	0.003	1.30 (1.00–1.67)	0.04	1.37 (1.05–1.79)	0.02
*HES5*	1.15 (0.95–1.40)	0.15	1.12 (0.92–1.36)	0.25	1.13 (0.92–1.38)	0.23
*CELF2*	1.34 (1.13–1.60)	0.0006	1.18 (1.00–1.40)	0.05	1.21 (1.02–1.43)	0.02
*EFCAB4B*	1.12 (0.95–1.32)	0.17	1.05 (0.89–1.22)	0.57	1.05 (0.90–1.23)	0.49

*Gene: Gene associated with the variably methylated regions (VMRs), most of the VMRs were located in the promoter region of the genes, *HR* hazard ratio, *CI* confidence interval

### Correlation with gene expression

**Fig. 4:**
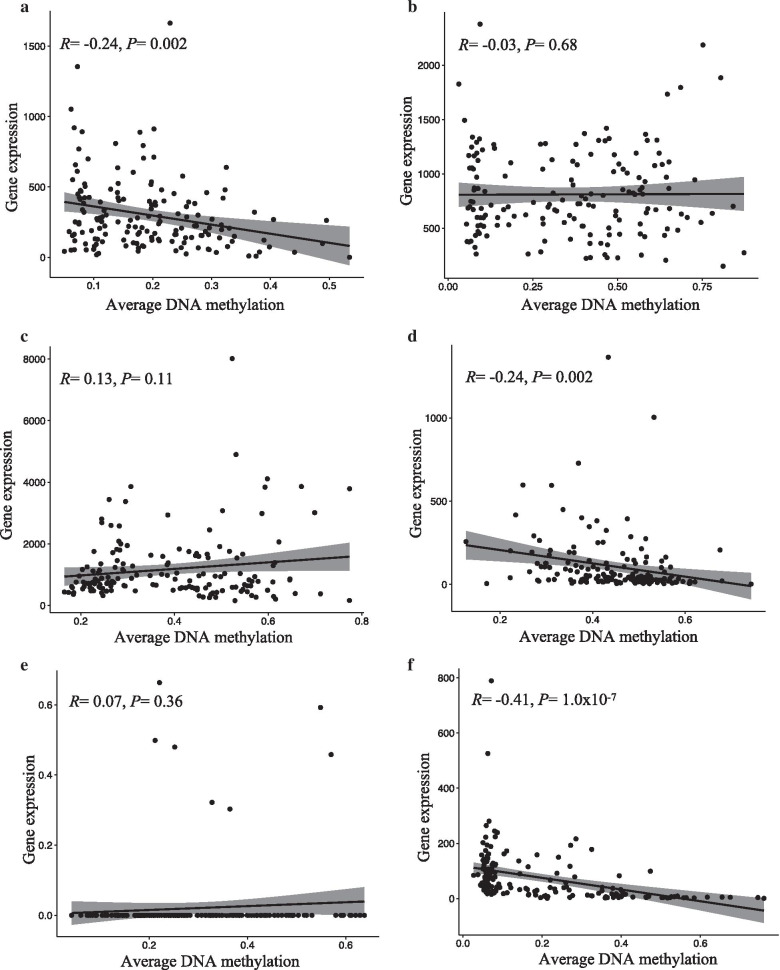

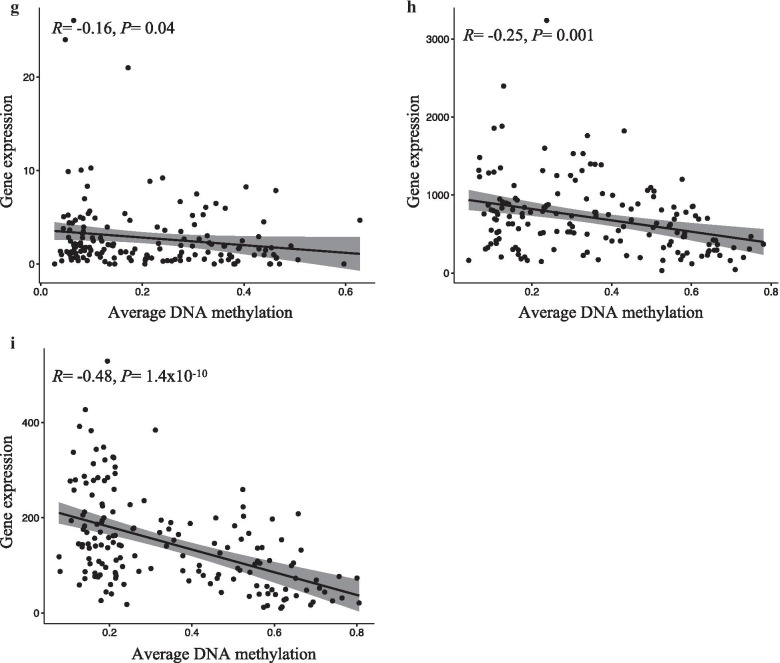
Correlation between methylation levels and gene expression. The graphics show the correlation between average DNA methylation (on the x-axis) and gene expression (on the y-axis) levels of invasive lobular breast cancer (ILBC) cases at the corresponding genes associated with nine of ten strongest variably methylated regions (VMRs) with available gene expression data in The Cancer Genome Atlas (TCGA) in the invasive lobular breast cancer (ILBC) cases in TCGA dataset: **a** *ISM1*, **b** *APC*, **c** *TMEM101*, **d** *ASCL2*, **e** *NKX6*, **f** *HIST3H2A*, **g** *HES5*, **h** *CELF2*, **i** *EFCAB4B*

A relatively strong negative correlation between DNA methylation and gene expression was observed for six of the nine tested VMRs in TCGA (Fig. 4). These included *EFCAB4B* (*R* = − 0.5, *P* = 1.4 × 10^−10^), *CELF2* (*R* = − 0.25, *P* = 0.001), *HIST3H2A* (*R* = − 0.41, *P* = 1 × 10^−7^), *ASCL2* (*R* = − 0.24, *P* = 0.002), *ISM1* (*R* = − 0.24, *P* = 0.002) and *HES5* (*R* = − 0.15, *P* = 0.04). No or slightly positive correlation between DNA methylation and gene expression levels was observed for *APC*, *TMEM101* and *NKX6*. The feature-by-feature analysis of correlations with gene expression was very consistent with the analysis using average methylation, virtually all associations being in the same direction, with only moderate variation in effect estimates (Additional file 3: Table S2).

## Discussion

We investigated the genome-wide DNA methylation pattern of ILBC tumours, with the aim of identifying methylation markers predictive of patient outcome. Scanning of the ILBC methylome revealed regions of variable methylation in ILBC tumours. The VMRs were primarily located in CpG island regions and were significantly enriched in pathways such as *breast cancer* (hsa05224), *pathways in cancer* (hsa05200), *hippo signalling pathway* (hsa04390), *Rap1 signalling pathway* (hsa04015) and *PI3K-Akt signalling pathway* (hsa04151). These pathways have previously been found to be dysregulated in cancer tissue [48–53]. Some of the key genes involved in the enriched pathways included *APC*, *DAPK1, BMP2 and CCND2. DAPK1* is an important regulator of cell apoptotic pathways [54] and *DAPK1* promoter hypermethylation has previously been reported in ILBCs with a potential role in tumour progression [45, 55]. *BMP2* is a member of the TGF-ß superfamily and is involved in cell proliferation and differentiation during tumour formation [56]. Promoter methylation of *BMP2* has been associated with breast cancer progression and drug resistance [57]. *CCND2* promoter methylation was previously reported to be a common event in breast cancer and have prognostic value [58]. We found a similar DNA methylation variability profile in TCGA dataset, in particular for the VMRs showing strongest variability in the MCCS.

Several previous studies have reported tumour DNA methylation to have prognostic value in cancer [59–64]. Methylation at many gene promoters has been reported to have independent prognostic value in breast cancer including *HOXA11* [65], *ESR1* and *PITX2* [66]*, HOXD13* [67] *CDH22* [68] *BRCA1* and *RASSF1* [69, 70]. Tumour DNA methylation and its prognostic significance has also been investigated for certain breast cancer subtypes, in particular gene expression-based subtypes. Thomas et al. (2017) used hierarchical clustering based on DNA methylation to further segregate luminal A tumours into two subgroups and found that the subgroup with lower relative methylation showed better prognosis [71], similar to the findings of our study. Another study using whole-genome methylation sequencing stratified triple-negative breast cancers into three methylation-defined clusters and found the hypomethylated cluster to show better prognosis compared with the other two highly methylated clusters [72], also consistent with our results. However, to our knowledge, no study has reported on the overall tumour methylation variability in ILBC and tested the potential for the variably methylated regions to be used as prognostic markers. The assessment of VMRs was genome-scale but only the highest ranking VMRs were tested for their association with survival. Although many of the tested VMRs showed a significant association with overall survival, there could be other VMRs or individual CpG sites for which methylation is associated with survival. We found that promoter hypermethylation at *APC*, *TMEM101* and *HCG4P3* was associated with shorter overall survival in the MCCS after adjustment for age and tumour stage. The results in TCGA were largely consistent with the MCCS, although associations generally appeared stronger; this might suggest that the prognostic value of these DNA methylation markers is greater for women with more advanced ILBC. In the pooled analysis, DNA methylation at four genes (*APC, TME101, HCG4P3* and *CELF2*) was associated with shorter overall survival. All the highest-ranking VMRs had an average methylation level below 0.5, and the direction of association with survival was virtually always positive, which indicates that methylation gains (i.e. loss of the normal hypomethylation state) were associated with worse survival. *APC* is a well-known tumour suppressor gene and this finding is in agreement with previous reports [73, 74]. Debouki et al. (2017) found a significant correlation between *APC* promoter methylation and aggressive behaviour of both non-familial and familial breast cancer in the Tunisian population [73]. The association of *APC* promoter methylation with reduced survival has also been reported for other cancer types, such as non-small cell lung cancer [75] and prostate cancer [76, 77]. CELF2*,* an RNA binding protein involved in alternative splicing, has also been reported to be involved in breast cancer growth and progression. Piqué et al*.,* (2019) found that *CELF2* promoter methylation led to a loss of *CELF2* expression that had a growth promoter effect in breast tumours. They also found that *CELF2* promoter methylation was associated with worse patient outcome [78]. In TCGA data, we found a strong, negative correlation between *CELF2* promoter methylation and the gene expression levels. TMEM101 is a transmembrane protein that has been shown to activate NF-kappa-beta signalling pathways. There is to our knowledge no previous literature suggesting a role of *TMEM101* promoter methylation in relation to cancer progression/survival. *HCG4P3* is also known as HLA complex group 4 pseudogene 3, and there is to our knowledge no record of this gene being involved in cancer.

The main limitation of this study was the relatively small sample size that limited our analysis to all-cause death as an endpoint. The MCCS and TCGA data had different characteristics in terms of their study design and sample variation. The two studies had different follow-up times, and TCGA data had more young women and generally higher tumour stage (Table 1). Our findings for both the VMR and survival analysis were nevertheless consistent across the two studies. We considered the main factors that we thought could impact methylation profiles in tumours and ILBC survival, i.e. age and stage. Factors such as smoking, alcohol consumption or diabetes, and perhaps family history (via underlying genetic sequence) likely play some role, but it is presumably less important, so we did not include them in the analysis. These variables are not systematically collected with precision (questionnaires) in the clinical setting. In this context, our study identified methylation biomarkers, and it is likely that many factors worthy of investigation (genetic and lifestyle and environmental) play a role in explaining the observed associations. Finally, while we identified a large number of regions across the ILBC genome that showed substantial variable methylation pattern, only the strongest ten VMRs were tested for association with survival to minimise the multiple testing burden. If replicated by other studies, the methylation markers identified in our study may contribute to the development of molecular signatures for enhanced prediction of ILBC survival.

## Conclusions

Our study indicates that methylation levels at the most variable regions across the genome may explain differences in tumour prognosis within the ILBC subtype. We identified *APC*, *TMEM101*, *HCG4P3* and *CELF2* promoter methylation as possibly relevant prognostic biomarkers for women with ILBC. Further studies are required to confirm our findings and to assess their utility in a clinical setting.

## Methods

### Study samples

The samples included in this study were obtained from the Melbourne Collaborative Cohort Study (MCCS) [79]. The MCCS was set up in 1990 with the aim of investigating the role of diet and lifestyle in cancer and other diseases. Between 1990 and 1994, 41,513 participants, aged 40–69, were recruited in the study, and baseline information on lifestyle, health and diet was collected through interviews. Women with ILBC included in this study were diagnosed between 1993 and 2011, based on the International Classification of Diseases for Oncology (ICD-O) codes 8520 (73%), 8522 (26%) and 8500 (1 case). Clinical and pathological characteristics of the study participants are listed in Table 1.

### Endpoints

Incidences of cancer cases and deaths in the MCCS participants are regularly updated by linkage to the Victorian and national cancer and death registries, which are considered to be virtually complete. The latest linkage was completed on 31 March 2017 and death data were considered to be complete up to 31 December 2016. Overall survival was defined as the time (in years) from breast cancer diagnosis to death (from any cause) or end of follow-up.

### DNA extraction from formalin-fixed paraffin embedded breast tumour tissue

Pathology material related to each ILBC case had previously been retrieved from the diagnostic service laboratory and reviewed by qualified pathologists. Unstained sections had been prepared and stored desiccated at 4 °C for up to 20 years. DNA extraction was conducted as described in Wong et al. [80]. Briefly, the tumour areas most suitable for macrodissection were identified by a qualified pathologist using the WHO classification of tumours of the Breast Criteria (WHO Classification of Tumours of the Breast (2012). 4th edn. International Agency for Research on Cancer (IARC), Lyon) [81] and recorded by directly marking up representative H&E stained sections. An average of two corresponding 3um methyl green stained FFPE sections were macrodissected as described in Wong et al. [82], and DNA was extracted using the QIAamp DNA FFPE protocol.

Tumour purity was estimated using the *R* tool *InfiniumPurify* [83] that takes methylation beta-values of the tumour samples and uses the methylation levels of pre-selected informative differentially methylated CpG sites (iDMCs) identified from TCGA data (when data from normal-adjacent tissue are not available) to estimate tumour purity for each tumour sample by density evaluation of Gaussian kernel. Tumour purity estimates, obtained as the proportion of tumour cells in each sample, were high, ranging from 37 to 88% across samples; 88% of the samples had an estimated tumour purity greater than 50%.

### Genome-wide DNA methylation profiling

Genome-wide DNA methylation was measured using the HumanMethylation450K (HM450K) BeadChip array (Illumina). For each sample, a total of 300–500 ng of tumour DNA was bisulfite-converted using Zymo Gold EZ-DNA kit (Irvine, CA) and restored using the DNA Restoration Kit as per the manufacturer’s instructions (Illumina, CA, United States). Sample DNA quantity was assessed using an in-house modified quality control protocol [80]. Samples that passed the final quality check were run on the HM450K array (Illumina) according to manufacturer’s instructions.

### Data pre-processing and normalisation

Raw intensity files (IDAT files) were imported into the R computing environment using the Bioconductor package *minfi* [84], and all samples were pre-processed and normalised together. Data quality was first evaluated by assessing the detection *P*-value, which was obtained for every CpG site in every sample. Samples with an average detection *P*-value > 0.01 were considered poor quality and were removed from further analysis. CpG probes with a detection *P*-value > 0.05 in at least one sample were considered unreliable and were removed from further analysis. Data were normalised using the *minfi* functional normalisation (FNORM) method to correct for both within-array (technical bias between type I and type II probes) as well as between-array unwanted variations [85]. After data pre-processing and normalisation, a total of 449,005 CpG sites remained for analysis. Beta-values (ratio of the methylated probe intensity and the sum of methylated and unmethylated probe intensity) and *M*-values (log_2_ beta-value) were calculated. *M*-values were used in all statistical analyses, while beta-values were used for data exploration and visualisation, as suggested in [86].

### TCGA data

Raw DNA methylation data (IDAT files) for 168 ILBC cases were downloaded from the TCGA legacy database (Study Accession: phs000178) using the R package *TCGABiolinks* [87]. Methylation data were pre-processed and normalised similarly to the MCCS, and methylation values (beta-values and *M*-values) were calculated for 168 ILBCs at 440,380 CpG positions across the genome. Survival data were retrieved for 159 (95%) ILBC cases. Gene expression data in the form of normalised counts (RNA sequencing-Illumina Hi-Seq) were retrieved for 159 (95%) ILBC cases. Cases of ILBC in the TCGA dataset were diagnosed between 1992 and 2013. Clinical characteristics of the TCGA samples are listed in Table 1.

### Statistical analysis

#### Variable methylation analysis

Variable methylation analysis was performed using the *DMRcate* package in R [88]. To identify the variably methylated regions (VMRs), the variance of *M*-values was computed across 130 ILBCs in the MCCS, and Gaussian smoothing was applied to the resulting per-CpG-site test statistics using the default *DMRcate* options. *DMRcate* uses the method of Satterthwaite to smooth test statistics and derive respective *P*-values. Nearby significant CpG sites were collapsed in clusters using a bandwidth of 1000 base pairs (bp). The clusters that showed the highest variability in DNA methylation (i.e. regions with a minimum adjusted *P*-value (minfdr) of less than 10^−8^) were defined as the VMRs. There were 396 solo CpGs that were not included in the VMR calculation. This analysis was replicated using the 168 ILBC samples from TCGA.

#### Gene set enrichment analysis

Gene set enrichment analysis was performed on all the genes associated with the VMRs using the web-based tool *Metaspace* using the default settings [89]. Pathway and gene set enrichment analysis were carried out using the KEGG Pathway database [90]. All genes in the human genome were used as the enrichment background. Pathways and biological terms with a *P*-value < 0.01, a minimum count of 3 and an enrichment factor > 1.5 (the ratio between the observed counts and the counts expected by chance) were selected and grouped into clusters.

#### Survival analysis

Survival analyses were undertaken for the ten most variably methylated regions identified across the MCCS ILBC samples. Follow-up started at the date of diagnosis and ended at the date of death or end of follow-up, whichever came first. Cox proportional hazards regression models were used to calculate hazard ratios (HRs) and 95% confidence intervals (CI) for the association between DNA methylation levels (*M*-values) and risk of death. Three models were fitted: (1) univariable, with DNA methylation as a crude predictor, and multivariable, (2) with additional adjustment for age at diagnosis and (3) with adjustment for age at diagnosis and tumour stage. For each VMR, the methylation level was defined as the average methylation value across all CpG sites covering the VMR. The same analysis was carried out using the 168 ILBC samples from TCGA. Survival analyses were undertaken using the R package *Survival* [91]. HRs from the two individual studies were then pooled using fixed-effects meta-analysis with inverse variance weights.

#### Association with gene expression

To test if DNA methylation correlated with gene expression at the ten strongest VMRs (identified in the MCCS), we assessed the correlation between average methylation levels (average *M*-values for all CpGs covering a VMR) and gene expression levels using Pearson’s correlation; we used matching gene expression and DNA methylation data available in the TCGA dataset for nine of the ten strongest VMRs. The correlations with gene expression were also assessed for individual CpG sites of each VMR.

## Supplementary information

**Additional file 1: Figure S1.** Relation between the number of CpGs related to each VMR and the VMR ranking

**Additional file 2.** List of variable methylated regions identified in ILBC.

**Additional file 3.** Correlation between DNA methylation and gene expression (feature-by-feature analysis).

## Data Availability

The datasets used and/or analysed during the current study are available from the corresponding author on reasonable request.

## Abbreviations

CI: Confidence interval
ER: Oestrogen receptor
FFPE: Formalin fixed paraffin embedded
FNORM: Functional normalisation
HM450K: HumanMethylation 450K
HER2: Human epidermal growth factor receptor 2
HRT: Hormone replacement therapy
HR: Hazard ratio
ILBC: Invasive lobular breast cancer
ICD-O: International Classification of Diseases for Oncology
IDBC: Invasive ductal breast cancer
MCCS: Melbourne Collaborative Cohort Study
MRI: Magnetic resonance imaging
PR: Progesterone receptor
TCGA: The Cancer Genome Atlas
VMR: Variably methylated region
