# Supplementary material for: Association of variably methylated tumour DNA regions with overall survival for invasive lobular breast cancer
Source: Clin Epigenetics. 2021 Jan 18;13:11. doi: 10.1186/s13148-020-00975-6 (PMC7814464; doi:10.1186/s13148-020-00975-6)
Supplement: Supplementary file 1 — Additional file 1: Figure S1. Relation between the number of CpGs related to each VMR and the VMR ranking [file 13148_2020_975_MOESM1_ESM.docx]

**Additional file 1: Figure S1. Relation between the number of CpGs related to each VMR and the VMR ranking**

The graphic shows the distribution of total number of CpG positions related to the variably methylated regions (VMRs), n=2,771, identified within invasive lobular breast cancer (ILBC) samples in the Melbourne Collaborative Cohort Study (MCCS). The ranks of the VMRs are shown on the x-axis and the number of CpG positions related to each VMR is shown on the y-axis and represented by the colour gradient as indicated in the legend on the right side of the plot.
